# Treatment of chronic osteomyelitis with antibiotic-impregnated polymethyl methacrylate (PMMA) – the Cierny approach: is the second stage necessary?

**DOI:** 10.1186/s12891-021-04979-y

**Published:** 2022-01-06

**Authors:** Noam Bor, Eytan Dujovny, Barak Rinat, Nimrod Rozen, Guy Rubin

**Affiliations:** 1 Orthopedic Department, Emek Medical Center, Afula, Israel; 2 Faculty of Medicine, Technion, Haifa, Israel

**Keywords:** Chronic osteomyelitis, Cierny-Mader, PMMA, Antibiotic cement spacers, Single stage treatment

## Abstract

**Background:**

Chronic osteomyelitis is a challenge for orthopedic surgeons. Most patients with osteomyelitis receive two-stage management according to Cierny-Mader. The first stage includes radical debridement and insertion of an antibiotic-impregnated cement spacer (ACS) (beads, rods, nails, or blocks) into the bone defect. The second stage is performed 6–8 weeks later, when the spacer is removed and a cancellous autograft is placed within the bone defect. The possibility of ACS as definitive management for osteomyelitis, avoiding the second stage, is presented.

**Methods:**

Sixteen patients with osteomyelitis received radical debridement and insertion of an ACS in all forms into the bone defect as a definitive management. In 8 patients, the tibia was infected, 4 had femur infection, 2 humerus, 1 fibula, and 1 ankle. The mean age at the time of the first stage of reconstruction was 49 years (range, 13–71 years). According to the Cierny-Mader classification, 1 patient was C-M IA, another was IB, 7 IIIA, 6 IIIB, and 1 was 4A. All B hosts had systemic illnesses. The mean follow-up period was 6 years (1.5–16 years).

**Results:**

No patient exhibited radiographic evidence of excessive bone loss. Signs of recurrence of osteomyelitis were not noted in any of the patients, and no fractures had occurred by the last follow-up.

**Conclusion:**

Our study suggests that a proportion of patients with planned retention of ACS appear to function well without requiring further surgical intervention, especially in elderly or vulnerable patients.

## Introduction

The treatment of chronic osteomyelitis is a challenging problem for the treating surgeon. The Cierny-Mader (C-M) classification, published in 1984, is based on the anatomy of the bone infection and physiology of the host. Cierny-Mader staging allows stratification of long-bone osteomyelitis and permits the development of comprehensive treatment guidelines for each of the stages [1].

C-M therapy for osteomyelitis is a two-stage approach: Adequate drainage, debridement, and obliteration of dead space are performed at the first stage, while antibiotic impregnated acrylic beads are used to sterilize and temporarily maintain the space. The types of antibiotics are depending on the wound flora which are sensitive to the antibiotic mixed with the cement. Aminoglycosides and vancomycin are common choices for local delivery because of their broad spectrum of activity and thermal resistance [1–15]. Within 4–6 weeks, the second operative stage is performed. The beads must be removed and replaced with a cancellous bone graft [1, 3–5, 7, 8, 10–12, 15].

The delivery of antibiotics locally can be executed by different antibiotic cement spacers (ACSs) in addition to antibiotic-impregnated acrylic beads: cement blocks, or intramedullary custom-made cement rods, all of which need to be removed. Alternatively, antibiotic cement-coated locked intramedullary nails can remain in the medullary canal indefinitely after control of the infection [16].

The purpose of this study was to evaluate the outcome of using ACSs in the form of beads, rods, intramedullary nails, or blocks as definitive management for the treatment of osteomyelitis.

## Patients and methods

We retrospectively reviewed the charts of 27 patients with chronic osteomyelitis who were treated in our institution using the described technique in the last 20 years. Seventeen of the 27 patients received only the first stage of treatment. One of these patients was excluded from the study because of short-term follow-up.

The diagnosis of infection was made according to clinical, laboratory, imaging, microbiological, and pathohistological features. All patients were evaluated by radiographs, either magnetic resonance imaging (MRI) or computerized tomography (CT), and some patients underwent three-phase Tc and indium-labeled leukocyte scintigraphy as part of the preoperative planning. Pain, swelling, and wound drainage were all indicative of infection. The patients were classified physiologically according to the Cierny-Mader criteria as A, B, or C hosts. The extent of osteomyelitis was classified anatomically as Types I through IV. The anatomical classification was then combined with the physiological class to designate the clinical stage of the patient [7, 8, 12]. Laboratory tests included white blood cell counts, erythrocyte sedimentation rate (ESR), C-reactive protein (CRP) levels, electrolytes, and infectious disease markers.

In the first and only stage involved in treating the bone infection, all previously inserted hardware was removed, tissue samples were obtained for microbiological analysis, and thorough soft tissue and bone debridement using special burr drills were performed on all patients. If no culture was available, one gram of vancomycin and one gram of gentamycin were mixed into the cement (40 g). If culture was available, based on knowledge from previous cultures taken from drained sinus for example, the cement was mixed with proper antibiotics the isolated pathogens are sensitive. The most commonly isolated germs were the *Staphylococcus aureus* and coagulase negative Staphylococcus species. Systemic antibiotics were delivered immediately after surgery. An accurate biological assesment included tissue and fluid cultures for aerobic, anaerobic acid fast bacilli, and fungal organisms. At the time of initial debridement/biopsy, Gram stain results was recorded as well. Initially cefazolin intravenously or broad-spectrum antibiotics were used empirically before the results of sensitivity came out (usually within 48 h) and were subsequently modified according to the culture and sensitivity results. After 2 weeks of intravenously treatment, it was changed to oral antibiotics according to the sensitivity results, while CRP, ESR, and routine blood test were regularly monitored. The antibiotic treatment was continued for a minimum of 6 weeks or until the ESR and CRP had returned to normal level.

As early as the third or fourth day after the surgery, all patients were encouraged to walk with partial or either full weight bearing using crutches. At the latest follow-up all patients were walking normally with full weight bearing.

## Results

We treated 16 patients (13 men and 3 women) with osteomyelitis with a one-stage technique. All patients were treated by the senior author (Table 1). Of these 16 patients, 8 had tibial infections (2 proximal, 2 shaft, and 4 distal), 4 had femur infections (3 distal and 1 shaft), 2 humerus (proximal and distal), 1 fibula (shaft), and 1 ankle. Ten patients had posttraumatic osteomyelitis, two had a history of hematogenous osteomyelitis, and four developed osteomyelitis following elective procedures. The mean age at the time of the first stage of reconstruction was 49 years (range, 13–71 years).

**Table 1:** Patient demographics

Patient	Age at surg. (yrs) /Gender	Cirny-Madar Stage	Bone Segment: PM D DM	Etiology of Infection	Microorganism	Duration of infection (yrs)	Outcome: Recurrent infection	ACS Type	F-U (yrs)
1	M/46	3A	Tibia R - DM	Open #	NC	25	No	Cement block	14
2	M/47	3B (Diabetes)	Femur Lt - DM	Open # - MVA	SA	18	No	I.M Rod + beads	14
3	M/15	3A	Tibia Rt PM	Open # MVA	PA	1	no	Beeds	16
4	F/67	3B (Diabetes)	Tibia Rt DM	Pin tract OM following deformity correction	SA	8	no	Cement block	10
5	M/13	1B (MMC)	Tibia Rt D	deformity correctio Int. Fix. Plating. NU	SA	0.5	no	Cement rods	7
6	M/71	3B (Diabetes)	Tibia Rt PM	Pin tract OM. # tibial Plataou. Ilizarov EF	SA	1	no	Cement block	1.5
7	M/65	3B (Diabetes)	Tibia Rt DM	# Open Pilon	NC	3	No	Cement Block	3
8	M/44	3A	Femur Rt DM	# Gun shot. EF → Plating	NC	2	No	Cement I. M Nail	2
9	F/59	3B (U.C)	Tibia Rt D	Abscess → Bacteremia	SM	0.5	No	Two Cement Rods	7
10	M/54	1A	Femur Rt D	Septic Knee → bacteremia	SA	1	No	Cemet I. M Nail	4
11	M/67	3B (CA of Colon)	Tibia Rt PM	ORIF for NU (s/p HTO for MU post opened #)	NC	5	NO	Cement Block	5.5
12	M/22	3A	Humerus Lt PM	# Per cutaneous Kirshner wires OM	SA	3	No	Cement Block	3
13	M/65	3A	Ankle Rt	# Open Pilon→ Ankle fusion (IM nailing)	PA	1	No	Cemented Ilizarov Rod	2.5
14	M/56	4A	Fibula LT D	# Conservative treatment	SA	5	No	Cement Rod	2
15	M/21	3A	Humerus Rt D	# Open Fracture (ORIF)	PR	3	No	Cement Block	1.5
16	F/64	3A	Femur Rt D	s/p Varus Osteotomy - Plating	SA	0.5	No	Cement Block	7

*M* male, *F* female, *UC* ulcerative colitis, *CA* cancer, *PM* proximal metaphysis, *D* diaphysis, *DM* distal metaphysis, *MVA* motor vheicle accident, *OM* osteomyelitis, *MMC* myelomeningocele, *EF* external fixation, *I. M* intramedullary, *ORIF* open reduction and external fixation, *NU* nonunion, *MU* malunion, *HTO* high tibial osteotomy, *SA Staphylococcus Aureus*, *PA Pseudomonas Aeruginosa*, *SM Serratia Marcescens*, *PR* Provedencia Rettgeri, *NC* not cultured

On the basis of the C-M classification, 1 patient was C-M IA, another was IB, 7 were IIIA, 6 were IIIB, and 1 was 4A. All B hosts had systemic illnesses.

The delay between the occurrence of bone infection and the treatment ranged from 0.5–25 years (mean, 5 years).

**Fig. 1:**
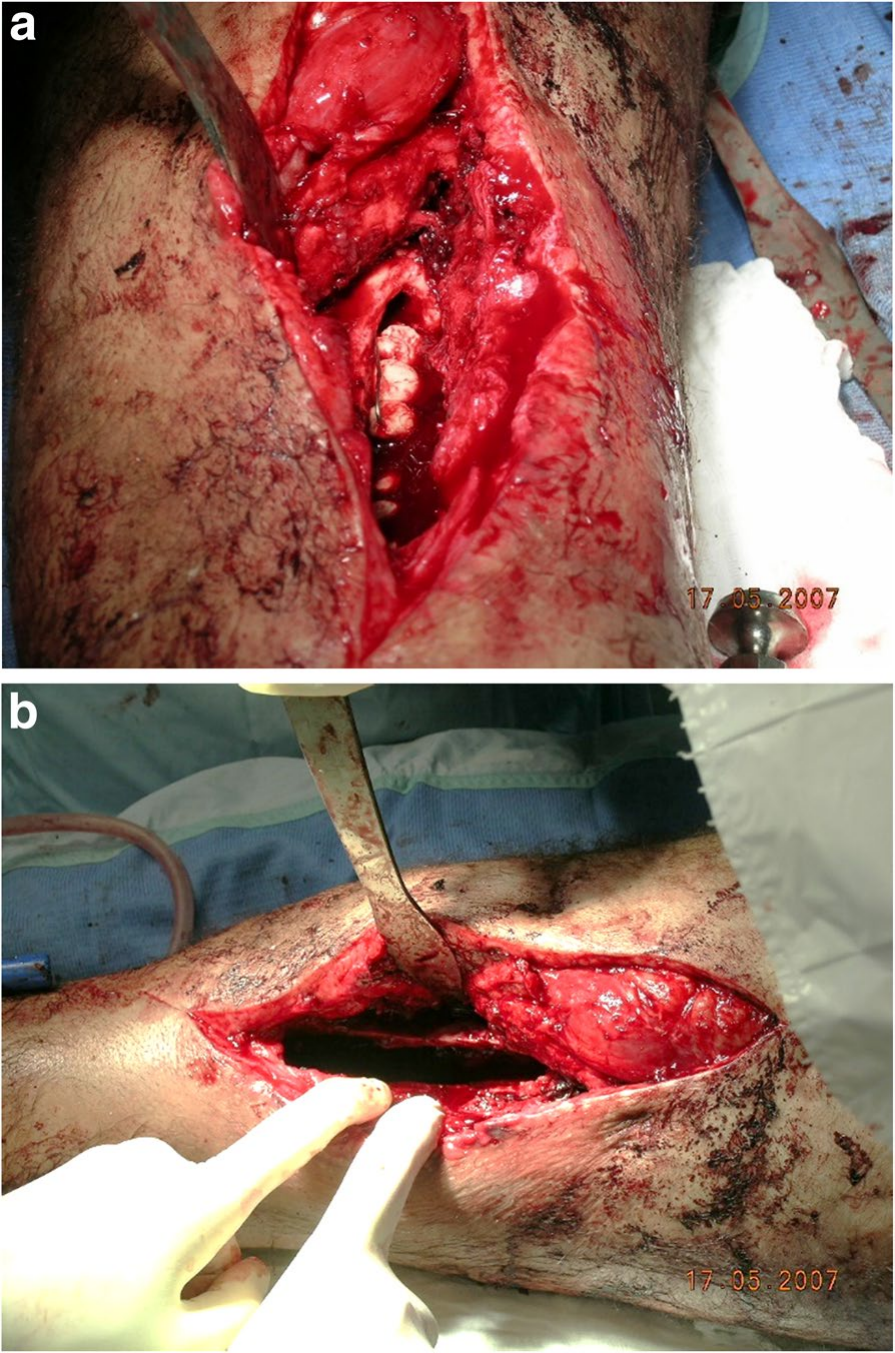
**a**, **b** Cierny 3A – femur/chronic OM for 8 years; operation year: 2007; debridement and insertion of PMMA – AB and antibiotic rod

**Fig. 2:**
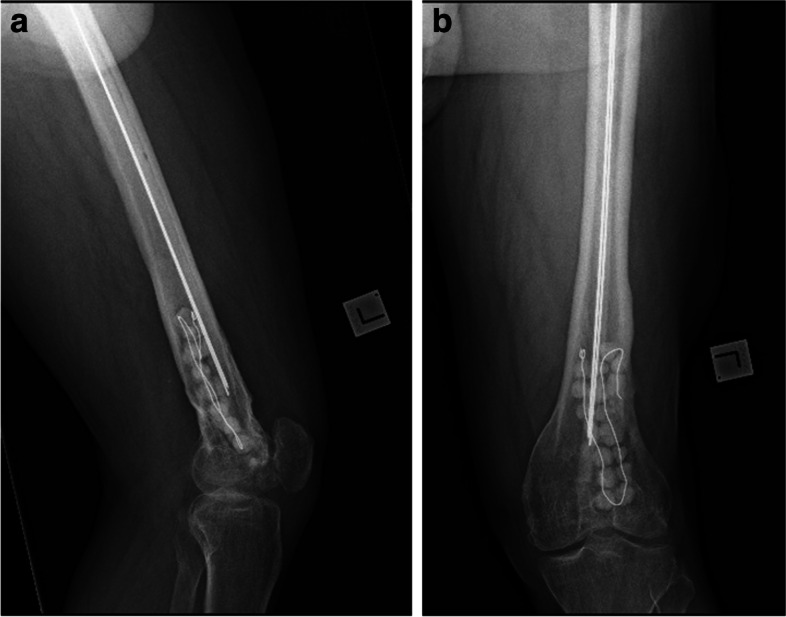
**a**, **b** Eleven-year follow-up (retained antibiotic beads and cement rods)

**Fig. 3:**
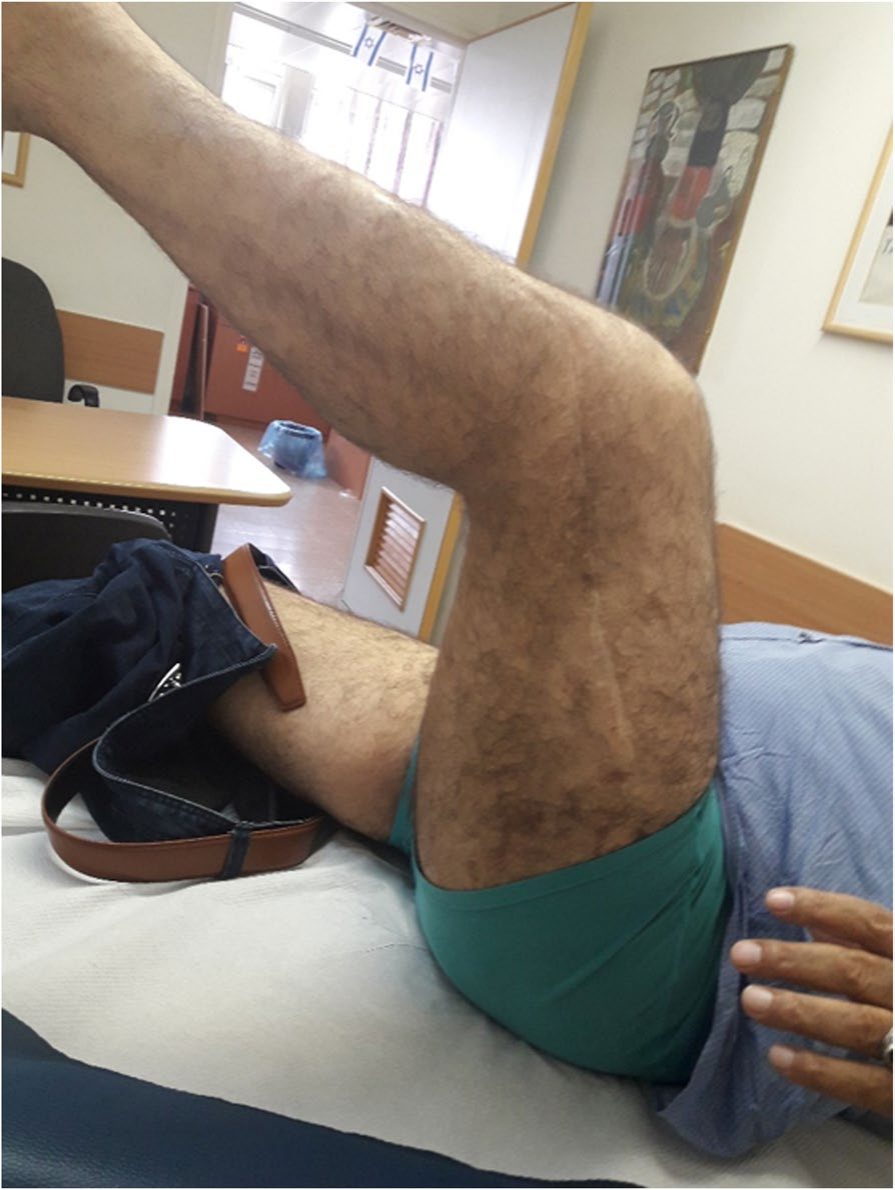
Eleven-year follow-up – no signs of infection; cemented rod and beads not removed

As for the second and third patients in this series,a second stage was considered, however, failed attempt in removing the antibiotics implants was recorded. In patient No. 2 (Figs. 1, 2 and 3), a surgical trial to remove the intramedullary cement rod and beads from the femoral canal was technically impossible; only a few of the cement beads were removed. In patient No. 3, only some of the antibiotic cement beads were removed, as they were stuck in thick fibrotic soft tissue.

The various types of antibiotic cement spacers included intramedullary nails in two patients, custom-made cemented rods plus beads in one patient, custom-made cemented rods in four patients, antibiotic beads in one patient, and antibiotic blocks in eight patients. Polymethyl methacrylate (PMMA) bone cement (Smith & Nephew, TN, USA) was used in all patients. Additional sensitive antibiotics were added to the powder before mixing the powder and the liquid.

A satisfactory result was defined as no drainage or open wound and no clinical, radiographic or laboratory evidence of infection. At the latest follow-up, ranging from 1.5 to 16 years (average, 6 years), none of the patients exhibited radiographic evidence of excessive bone loss, signs of recurrence of osteomyelitis, or fractures. All patients had no drainage, pain or signs of inflammation, and all laboratory parameters were normal.

## Discussion

Our study on 16 patients proves the theory that the second stage of the Cierny-Mader approach to chronic osteomyelitis, in certain circumstances, can be avoided, and no complications are due to come out by retaining the ACA for long standing.

In 1984, Cierny and Mader published a unique classification system for chronic osteomyelitis, taking into account the importance of the immune competency and physiological ability of the host to affect healing, and the anatomic nature of the disease, introducing the two-stage technique [1].

The technique has the following disadvantages: The polymethyl methacrylate (ACS) is required to be removed. Obviously, there are independent medical and surgical risks by performing an additional operation, as well as costs both to the patient and health care system (24). ThePMMA can also offer a substrate for bacterial colonization when the release of antibiotics declines over time and finally becomes ineffective. However, the main advantage of this technique is thehigh local antibiotics concentrations achieved, the risk of systemic toxicity is minimized, the long-term exposure to intravenous antibiotics delivery, which is strongly related to the emerging threat of antibiotic resistance, is avoided [17].

Therefore, many surgeons were looking for effective one-stage procedure, using different antibiotic delivery systems which are not required to be removed, antibiotic-impregnated bioabsorbable bone substitutes. New biomaterials were introduced: Collagen fleece, an effective triphasic antibiotic release, and polyesters which offer a slower breakdown and some evidence of intracellular action [18]. Calcium-based carriers, including plaster of Paris, calcium sulfate [19], and calcium hydroxyapatite, allow tissue and bone ingrowth as they degrade. Insertion of tobramycin-impregnated calcium sulfate pellets recorded to be effective in a group of 12 skeletally immature patients with chronic osteomyelitis of the long bones [20]. Another recent study showed one-stage treatment of chronic osteomyelitis with bioactive glass S53P4 [21]. Other potential delivery systems are polyanhydrides, amylose starch, and composite carriers [18, 19].

The various biocompatible and bioabsorbable technologies are especially useful, if a planned secondary procedure for re-instrumentation, bone grafting, or soft-tissue reconstruction are not required. However, the biodegradable products have their drawbacks: (1) Not strong enough as load-bearing spacers; (2) Intrinsic chemical intolerances preventing the product(s) from hardening limiting the amount of mixing up antibiotics. (3) Inflammatory byproducts released during their degradation creating eventually wound seromas in as many as 20–28% of cases [22].

None of the patients in our study received second-stage reconstruction, they all, except for one, were of type 1 through 3 according to the Cierny-Mader classification, and retained the ACS. Following the first stage of treatment, all bone segments were stable enough, the mechanical integrity of the remaining bony segments in patients with Cierny-Mader type III was satisfactory, therefore, there was no need to perform any stabilization procedure or reinforcement of bone segments at a second stage.

Our method is not applicable in patients with Cierny-Mader type IV, where a considerable amount of bone segment is required to be removed during the first stage of treatment, major reconstruction is needed, performed during the second stage. Therefore, these patients must be treated according to the traditionally 2-stage treatment recommended by Cierny-Mader.

The only patient in our study with Cierny Mader type IV, the fibula was involved, a non-weight bearing bone, therefore a second stage with major defect reconstruction was not required. None of the ACS drawbacks described above were noted during long-term follow-up. None of the patients exhibited any sign of osteomyelitis recurrence.

The decision to avoid the second operative stage was also supported and encouraged by our favorable experience with the earlier two patients in this group, in whom a trial to remove the ACS failed. Three years of follow-up on these patients resulted in no complications.

To the best of our knowledge, only two reports referring to the long-term use of ACSs in patients with osteomyelitis in long bones are available: In a study by Xu-Sheng Qiu et al. [23], cement spacers were not removed following treatment for chronic osteomyelitis in eight patients (7 tibias, 1 calcaneus). After a mean follow-up period of 2 years, no signs of recurrence of osteomyelitis were noted in any of the patients. In another recent study by Frenando et al. [24], reporting on 37 patients who did not undergo antibiotic-impregnated PMMA bead removal, at long-term follow-up (range 6 months-5 years), there were no wound complications or recurrent infections.

Paley and Herzenberg treated infected intramedullary canals with antibiotic impregnated custom-made cement rods in nine patients. In one 70-year-old patient, the rod was not removed, and no signs of recurrent infection were noted 38 months later [13]. In our study, the follow-up was longer, and various types of cement spacers were used in comparison to other literature reports.

Our decision to avoid the second-stage operation according to Cierny-Mader is also based on the vast experience of joint replacement surgeons and the related literature regarding the use of PMMA bone cement. The use of cemented femoral stems is safe, increasing with the aging population for both elective and nonelective hip arthroplasty especially [25]. Choi et al. reported 18 patients with periprosthetic joint infection (11 hips, 7 knees) treated with retained prosthetic articulating spacers. At an average of 43.8 months (range, 13–78 months) of follow-up, no cement spacer-related complications were noticed [26].

Ferrao et al. [27] reported using ACSs as definitive management for postoperative ankle infection. At a 20.1-month (range, 6 to 62 months) follow-up, seven patients still retained their cement spacer with no complications.

According to our results, we believe that a second operation with all its risks can be avoided, especially in elderly or vulnerable host B or C patients. The use of absorbable biomaterials has its downsides, as reported above; in addition, they are expensive and not always available in the developing world.

## Data Availability

All data generated or analysed during this study are included in this published article.
